# Domestic

**Published:** 1841-06-26

**Authors:** 


### DOMESTIC.

*The British and Foreign Medical Review on* Dr. Paine's *Medical and Physiological Commentaries.*—A perusal of Dr. Paine's work has impressed us with a great respect for the author's industry and zeal; but we fancy that his mind must be deficient in one qualification, which is rather important to any one who sets up as a philosopher, (a title of which we should judge, by the heading of four of his essays, that the writer is rather ambitious,) namely, the capability of perceiving accurately the relations of ideas. This notion we have formed from the multitude of instances of loose and incoherent reasoning, of contradictory statements, and of misinterpretations of the opinions of others, which we have detected in his treatises. To bring a single objection against the assertion of another—whether that assertion be the statement of a fact or the enunciation of a principle—seems in his mind quite sufficient to overturn it. To show that a certain law is not capable of explaining all the phenomena of a given action, appears to be regarded by him as quite sufficiently proving that the law had no bearing on the action. Now, any one who has given the slightest attention to the science of life—Biology—must be aware how little any phenomena exhibited by living beings can be attributed to the undisturbed operation of any single law, and how constantly the physiologist and pathologist are obliged to make allowance for circumstances which interfere with the operation of the simplest and best understood principles. But, it may be asked, does not the author lay himself open to similar attacks? Are his doctrines more securely defended than those of others? In his own estimation they certainly are; but we must take leave to differ from him. The foundation of his system is the attribution of every phenomenon which cannot be *fully* explained in any other way, to the undivided operation of the "vital forces;" a sort of "independent company," of which the "vital principle" is the commander. Any attempt to penetrate their ranks is useless; their shields form an impassable barrier, and their spears defy the invader. But ancient armour is of little avail against the resources of modern warfare; a few cannon-balls sweep the supports of these old-fashioned soldiers from under them, and the invaders boldly make their way into the guarded spot. To speak without metaphor, we consider that any attempt to check the course of inquiry, by thus attributing all unexplainable or unexplained phenomena to the immediate influence of that mysterious agent, the "vital force," is a decided retrogression in physiological science. For there can be no doubt that many of these phenomena are, to say the least, partly explicable on physical principles; and that those which result from the vital properties of organized bodies still take place under the government of fixed laws, which it is the legitimate province of the philosopher to investigate.

It seems to us as if Dr. Paine's mind—possessing, as it evidently does, much power, but, unfortunately, misdirected in the application of it—would have been much benefitted by that training in the school of physical science, which would have given him habits of more correct reasoning, and a clearer perception of the bearing of general principles. The latter with some persons is intuitive, by others it is acquired: from whatever source it arises, it is one of the chief characteristics of a truly philosophic mind. We cannot better illustrate the deficiencies and errors we have laid to Dr. Paine's charge, than by referring briefly to his "Appendix on the Microscope," (vol. i. p. 699.) The extended use of this instrument "threatens," in his opinion, "a subversion of physiological science." He forgets that, faithfully employed, it can only disclose *facts*. But facts are stubborn things. He quotes a number of instances in which microscopic observers have differed—as, for example, the size and form of the globules of the blood—the structure of the nervous tubules; but he does not tell us how completely, with instruments much improved during the last few years, and with increased knowledge of the circumstances which can affect the accuracy of the observation, all good microscopists are now agreed on these as upon a multitude of other interesting and important questions. "Finally," he concludes, "our microscopical philosophers would have it, that all nature may be ultimately resolved into microscopical *insects*. (!) The very rocks are composed of them—nay, the earth itself. . . . . *On weighing one of their shells*," he afterwards tells us, "it was found to be the 187,000,000th part of a grain;" a most unfair distortion of an *estimate* founded upon measurement of the size of these curious bodies, and the known weight of collections of them containing a number which may be calculated by their size. In upholding this ridiculous doctrine, he tells us that "Professor Hitchcock is alone in his glory in the western hemisphere." We are assured, however, on the authority of Dr. Paine, who does not mention having examined the objects for himself, that "the objects mistaken for fossil animalculæ are nothing but crystalline spiculæ of earthy or metallic substances." When Dr. Paine has taken the trouble to examine, as we and hundreds of others have done in this country, the living animalcules, and the beautifully organized siliceous cases which accumulate as a kind of mud at the bottom of the water in which they breed, and has compared these with similar remains, which, preserved in the most complete perfection, constitute the Polierschiefer of Bilin, or the mealy silicious earth used as food by the Laplanders, he may perhaps regret having hazarded his credit upon so rash an assertion.

---

INTERMENTS *in the City and Liberties of Philadelphia, from the* 12*th of June, to the* 19*th.*

| Diseases. | Adults. | Children. | Diseases. | Adults. | Children. |
|---|---|---|---|---|---|
| Amenorrhæa, | 1 | 0 | *Brought forward*, | 37 | 39 |
| Cancer, | 1 | 0 | Marasmus, | 0 | 5 |
| —— Stomach, | 1 | 0 | Measles, | 0 | 2 |
| —— Womb, | 1 | 0 | Mania a potu, | 3 | 0 |
| Casualty, | 1 | 0 | Old age, | 1 | 0 |
| Croup, | 0 | 3 | Palsy, | 2 | 0 |
| Congestion of | | | Scrofula, | 0 | 1 |
| Lungs, | 0 | 2 | Small pox, | 0 | 3 |
| —— Brain, | 1 | 1 | Spina Bifida, | 0 | 1 |
| Consumption of | | | Still-born, | 0 | 4 |
| the lungs, | 14 | 5 | Suicide, | 2 | 0 |
| Constipation, | 1 | 0 | Summer Com- | | |
| Convulsions, | 1 | 7 | plaint, | 0 | 7 |
| Diarrhœa, | 0 | 2 | Ulceration, | 1 | 0 |
| Dropsy, | 1 | 1 | Ulcerated sore | | |
| —— Head, | 0 | 2 | mouth, | 0 | 1 |
| —— Breast, | 2 | 0 | Unknown, | 0 | 1 |
| Disease of stom'h. | 0 | 1 | Violence, | 0 | 1 |
| Drowned, | 0 | 1 | Vomiting of blood, | 1 | 0 |
| Dysentery, | 0 | 1 | Worms, | 0 | 1 |
| Debility, | 1 | 0 | | — | — |
| Erysipelas, | 0 | 1 | Total, | 113—47 | 66 |
| Enlargement of | | | | | |
| heart, | 1 | 0 | Of the above, there | | |
| Fever, Catarrhal, | 0 | 2 | were under 1 year, | | 35 |
| —— Typhus, | 1 | 0 | From 1 to 2 | | 12 |
| —— Hectic, | 1 | 0 | 2 to 5 | | 10 |
| —— Scarlet, | 0 | 1 | 5 to 10 | | 6 |
| Hernia, | 1 | 0 | 10 to 15 | | 1 |
| Hæmorrhage | | | 15 to 20 | | 2 |
| from lungs, | 1 | 0 | 20 to 30 | | 7 |
| Inflammation of | | | 30 to 40 | | 17 |
| the Brain, | 0 | 1 | 40 to 50 | | 10 |
| —— Bronchi, | 0 | 1 | 50 to 60 | | 3 |
| —— Lungs, | 1 | 2 | 60 to 70 | | 2 |
| —— Bowels, | 0 | 5 | 70 to 80 | | 4 |
| —— Pleura, | 1 | 0 | 80 to 90 | | 3 |
| —— Peritonæum, | 2 | 0 | 90 to 100 | | 0 |
| Intemperance, | 2 | 0 | | | —— |
| | — | — | Total, | | 113 |
| *Carried forward*, | 37 | 39 | | | |

Of the above there were 9 from the alms-house, 17 people of colour, and 2 from the country, which are included in the total amount.

